# Estimating Three-Dimensional Body Orientation Based on an Improved Complementary Filter for Human Motion Tracking

**DOI:** 10.3390/s18113765

**Published:** 2018-11-04

**Authors:** Chunzhi Yi, Jiantao Ma, Hao Guo, Jiahong Han, Hefu Gao, Feng Jiang, Chifu Yang

**Affiliations:** 1 School of Mechatronics Engineering, Harbin Institute of Technology, Harbin 150001, China; chunzhiyi123@gmail.com (C.Y.); 17S108247@stu.hit.edu.cn (J.M.); 17S108251@stu.hit.edu.cn (H.G.); 17S108249@stu.hit.edu.cn (J.H.); cfyang@hit.edu.cn (C.Y.); 2 School of Electrical Engineering, University of New South Wales, Sydney 2033, Australia; hefu.gao@student.unsw.edu.au; 3 School of Computer Science and Technology, Harbin Institute of Technology, Harbin 150001, China

**Keywords:** inertial and magnetic sensors, orientation estimation, human motion tracking, complementary filter, Kalman filter

## Abstract

Rigid body orientation determined by IMU (Inertial Measurement Unit) is widely applied in robotics, navigation, rehabilitation, and human-computer interaction. In this paper, aiming at dynamically fusing quaternions computed from angular rate integration and FQA algorithm, a quaternion-based complementary filter algorithm is proposed to support a computationally efficient, wearable motion-tracking system. Firstly, a gradient descent method is used to determine a function from several sample points. Secondly, this function is used to dynamically estimate the fusion coefficient based on the deviation between measured magnetic field, gravity vectors and their references in Earth-fixed frame. Thirdly, a test machine is designed to evaluate the performance of designed filter. Experimental results validate the filter design and show its potential of real-time human motion tracking.

## 1. Introduction

Accurate orientation estimating plays an essential role in aerospace, robotics, navigation and healthcare-related applications. In healthcare-related applications, motion tracking is a key technology for analysis of rehabilitation treatment [1], fall detection [2] for the safety of elderly people, rehabilitation robots [3], and diagnosis for some muscular dystrophy diseases. To determine the orientation of human body, several motion-tracking technologies have been developed, including mechanical, LIDAR motion trackers, optical motion-tracking system, and inertial/magnetic motion-tracking system.

Mechanical motion trackers estimate [4] and track the orientation of human limbs using an exoskeleton where goniometers are mounted on the junction of skeletal linkages to measure joint angle. Exoskeleton-based motion tracker can provide force or haptic feedback. However, it suffers from axis misalignment and limited degrees of freedom (DOFs) of exoskeleton joints. In addition, the hulking volume and bad wearability of exoskeletons are other limitations in using mechanical motion trackers.

LIDAR motion trackers [5] use a 2-D LIDAR to detect the shape of a human body. By comparing it with the model database, body orientation and the distance between LIDAR transmitter and human body are estimated. Such system needs hefty computers to store data and run image-processing algorithms.

Optical motion-tracking system [6] estimates orientation through multiple cameras to track pre-designated points on moving limbs in a fixed working space. Similar to LIDAR motion trackers, this method needs hefty computers to store data and run image-processing algorithms. In addition, the needs of HD cameras and proper lighting conditions also limit its application in laboratory.

Compared with the off-line methods stated above, benefitting by advances in MEMS technology, tiny and low-cost inertial sensors called IMU enable the self-contained and wearable measurement of orientation. In addition, its capability of being attached on each segment of human makes it possible to independently determine its orientation relative to an Earth-fixed frame. The IMU-based inertial/magnetic motion-tracking system thus can be used to real-time estimate orientation without range limitation. To estimate 3-D orientation, a 9-axis IMU (Inertial Measurement Unit) that consists of a tri-axis accelerometer, a tri-axis gyroscope and a tri-axis magnetometer is required to provide a complete measurement of orientation relative to the direction of magnetic field and gravity. An orientation estimating algorithm that can fuse sensor information of IMU into an optimal orientation can be used as a functional component of any IMU-based motion tracking system.

In this paper, the design, implementation, and experiment of an automatic coefficient-tuning complementary filter are performed to estimate the 3-D orientation of human body segments described in the sensor-fixed frame with respect to the Earth-fixed frame. To do so, data from an IMU are used as input of the designed filter. In addition, the orientation is represented by quaternions for improving calculation efficiency and avoiding singularity. The filter compensates for the error caused by magnetic distortion and the assumption of little movement through continuously fusing the quaternion integrated from angular rate and the quaternion estimated by accelerometer and magnetometer measurements. A factored quaternion algorithm (FQA) is used to process magnetometer and accelerometer measurements to simplify the design of complementary filter. The fusion coefficient is auto-tuned based on the deviation between the measurements rotated by estimated orientation and the local reference vector at each sample point through a linear function. In addition, the function is predetermined using just a few sample points before fusing quaternions. To compare the accuracy and real-time calculating potential of the designed complementary filter, an adaptive Kalman filter is also introduced based on [7]. In addition, both Kalman filter and complementary filter we designed are validated by comparing their performance with other three complementary filter-based algorithms on a test platform.

The primary contributions of this paper are:

- A complementary filter whose fusion coefficient is auto-tuned by a predetermined function is designed for real-time human segment orientation tracking;
- An adaptive Kalman filter based on [7] is designed for online estimating the measurement convergence;
- The performance of two algorithms stated above is validated on an especially designed test platform to make a comparison with the performance of some previously existing algorithms.

The paper is organized as follows. Section 2 provides an overview of related work, and contrast that with the designed filter. Section 3 presents the design of the novel automatic-coefficient-tuning complementary filter and a brief description of designing a Kalman filter to have a comparison with our proposed method; Section 4 introduces experimental facilities and experimental results. Section 5 provides a summary and conclusion.

## 2. Related Work

Human motion tracking using inertial sensors which could be treated as an application of Wahba’s problem has been studied for many years. As opposed to algorithms that estimate the inclination of human body [8,9] and orientation relative to other limb segments to calculate joint angles, only algorithms that estimate 3-D orientation of a limb segment relative to an earth-fixed frame are introduced as related work. In this section, previous work of human body orientation estimation will be cataloged in three parts: simple integration from angular rate, vector observation and data fusion-based algorithms.

### 2.1. Angular Rate Integration

The simplest way of estimating orientation is to integrate the angular rate from an initial known rotation. As the angular rate measured by gyroscopes is directly integrated, the estimating of orientation is smooth even in rapid movements, which is given by:

(1) $$q_{t}=q_{t-1}+\frac{\Delta t}{2}\left[\begin{array}{c} 0\\ \omega_{t} \end{array}\right]⊗q_{t-1}$$

However, angular rate integration will lead to a drift due to the low-frequency noise in the measurement of gyroscopes. In addition, in most cases, the initial orientation of sensor-fixed frame cannot be known.

### 2.2. Vector Observation

Another simple way of estimating orientation is through vector observation, including TRIAD, QUEST [10] and FQA [11]. Vector observation provides an orientation estimate relative to a fixed Earth-fixed frame by measuring at least two vectors of local frame and comparing these vectors with the known positions of vectors in a fixed Earth-fixed frame. Formally, a rotation matrix R is intended to be found:

(2) $$r_{i}=Re_{i},\;i\in \left[1,n\right]$$

where $e_{1}$, …, $e_{n}$ are the set of vectors in local frame and $r_{1}$, …, $r_{n}$ are the set of reference vectors in the Earth-fixed frame. To solve this problem, TRIAD algorithm uses two nonparallel normalized measurement vectors as input. It constructs two triads of orthonormal unit vectors from two inertial measurements, which are gravity and magnetic field vectors in both Earth-fixed frame and sensor-fixed frame, to calculate the rotation matrix R. The TRIAD algorithm is a suboptimal algorithm that uses magnetic measurement without restriction, which will contribute to errors in two rows of rotation matrix due to the magnetic distortion.

The QUEST algorithm [10] is an optimal algorithm of estimating the rotation relationship between a sensor-fixed frame and Earth-fixed frame deriving from minimizing the loss function:

(3) $$L\left(R\right)=\frac{1}{2}\sum_{i=0}^{n}a_{i}∥W_{i}-RV_{i}∥$$

with respect to 3×3 rotation matrix R, where $a_{i}$ is nonnegative weights , $W_{i}$ is reference vector and $V_{i}$ is measurement vector. In addition, at least two unparalleled vector pairs $(W_{1}$, $V_{1})$ and $(W_{2}$, $V_{2})$, which refers to acceleration and magnetic field vectors in sensor-fixed frame and Earth-fixed frame, are used as input. However, due to the use of optimal method for minimizing loss function, codes should be iterated several times at each sample point which contributes to relatively low calculation efficiency.

Recently, in [12], Dung Phan et al. improve the QUEST algorithm by combining geometrical constraint of a human limb and change the rotation matrix R into a piecewise function of the human limb working space. While this improvement increases the accuracy of QUEST algorithm, it reduces the computing efficiency further and limits its application scenario.

The FQA algorithm, which will be introduced as the basis of this paper in the next section, is a geometrically intuitive orientation estimating algorithm. It produces a quaternion-based orientation estimate through three rotations while restricting the use of magnetic measurements to the rotation around vertical axis. Compared with TRIAD and QUEST, the advantages of FQA algorithm are its high calculation efficiency and restricted use of magnetic field vectors.

Vector observation could provide an absolute orientation estimate of sensor-fixed frame. However, because the observations of gravity and magnetic field are corrupted by the acceleration caused by segments’ movement and magnetic field distortion respectively, the orientation it estimates suffers from high-frequency noise.

### 2.3. Data Fusion-Based Algorithms

#### 2.3.1. Kalman Filter

To produce an accurate orientation estimate, the orientations estimated by angular rate integration and vector observation should be fused to compensate flaws of each other. Kalman filter has become the majority of 3-D orientation fusion.

Yun et al. [7] used an extended Kalman filter to fuse orientation quaternion estimated from QUEST and angular rate integration. It treated quaternion estimated from QUEST as measurements and used angular rate to construct the state equation of Kalman filter and then validated its performance using a 2-DOF tilt table. Makni et al. designed an adaptive Kalman filter that can online estimate the observation convergence matrix from the residual of accelerometer measurement update in [13]. Sabatini et al. designed an extended Kalman filter that could track 3-D orientation of human body in [14]. In this proposed method, the measurement convergence matrix is dynamically determined by thresholds of deviation between magnetic field and gravity vectors in earth-fixed frame and sensor-fixed frame. Roetenberg et al. [15,16] proposed a complementary Kalman filter treating gyroscope bias error, orientation error, and magnetic disturbance error as state variables, which works as a part of XSens MVN [17]. In this work, magnetic dip angle and magnetic flux were used as a measurement of magnetic distortion to dynamically calculate the standard deviation of Gauss white noise in magnetic disturbance model.

The common disadvantage of Kalman filter is the variety of its parameters which leads to a complex parameter adjustment process. In addition, the parameters need to be tuned again when the variance of Kalman filter changes.

#### 2.3.2. Complementary Filter

As a substitute of Kalman filter, complementary filter is much more computationally efficient. However, its fusion coefficient is too sensitive to be practically used. To solve this problem, Banchmann et al. [18] proposed a quaternion–based complementary filter introducing Gauss-Newton method to preprocess the acceleration and magnetic field vectors. However, Gauss-Newton method should be iterated at each sample point, which reduces the computing efficiency. In addition, in [19], Gallagher et al. described a complementary filter with lower complexity and similar accuracy to the algorithm presented by Banchmann et al. [18] with validation on a manipulator. However, the fusion coefficients of this algorithm need to be tuned manually according to signal properties of different application scenarios, which limits its availability. In [20], Wu et al. designed a two-layer complementary filter and tested the performance on an IMU-based platform. Quaternions estimated by acceleration and gyroscope are fused together in the first layer. Using a second complementary filter, the quaternion estimated by vector observation, which uses magnetic field and acceleration vectors, is fused with the output from the first layer.

More recently, Madgwick et al. [21] presented a real-time orientation estimating algorithm based on gradient descent method, which is widely known as the most accurate open source orientation estimating algorithm. It estimated the orientation of a sensor-fixed frame by minimizing the cost function shown as follow, which is then fused with quaternion integrated from angular rate using a complementary filter. However, this algorithm still suffers from the magnetic distortion a lot despite of its correcting algorithm.

(4) $$f_{mag}\left(q_{S}^{E}\right)=∥q_{S}^{E}⊗\left[\begin{array}{c} 0\\ m \end{array}\right]⊗{q_{S}^{E}}^{-1}-\left[\begin{array}{c} 0\\ N \end{array}\right]∥$$

(5) $$f_{acc}\left(q_{S}^{E}\right)=∥q_{S}^{E}⊗\left[\begin{array}{c} 0\\ a \end{array}\right]⊗{q_{S}^{E}}^{-1}-\left[\begin{array}{c} 0\\ G \end{array}\right]∥$$

where $q_{S}^{E}$ is a quaternion representing the orientation of the sensor-fixed frame relative to the Earth-fixed frame, m, a are vectors measured by magnetometer and accelerometer of IMU respectively, and N and G are local magnetic field vector and gravitational acceleration vector in the Earth-fixed frame respectively. In [22], Seel et al. calibrated the drift of angular rate integration in a control point of view by designing a proportional controller and correct the quaternion-based orientation through compared to magnetic field and gravity observations. Similarly, a complementary filter is designed to correct angular rate in a proportional-integral way and validated using a designed 1-DOF platform [23]. In this kind of algorithms, the coefficient of proportional controller, which is very sensitive, needs to be tuned based on the performance of experiments.

Other than the Kalman filter-based and complementary filter-based algorithms above, Yadav et al. [24] proposed a particle filter-based algorithm, which detects the corruption of magnetic field and acceleration by dip angle and acceleration vector norm, respectively. Although reducing the errors caused by magnetic distortion, the computational complexity of it is relatively high while the thresholds used to detect the corruption of magnetic field and acceleration should be tuned manually to fit different application scenario.

To summarize, the ideal method of estimating 3-D orientation is to compensate errors caused by limb-movement acceleration, magnetic distortion, and low-frequency noise integration, while relatively small computing resource is required. Due to orientations could be separately obtained by different signals, orientation estimating algorithm should dynamically weigh the credibility given to orientations estimated from acceleration, magnetic field, and angular rate with relatively low computational cost. In contrast with algorithms described above, this paper presents an especially designed complementary filter-based algorithm to track 3-D orientation of human-limb segments. This algorithm adopts two steps to estimate orientation. The first step is to determine a linear function whose dependent variable is the fusion coefficient of complementary filter and independent variable is the deviation between the measurements rotated by FQA quaternion and the local reference vectors. In addition, the next step is to use this predetermined function to dynamically fuse the quaternion integrated from angular rate and the FQA quaternion which is estimated using magnetic field and gravity vectors. By using this algorithm, errors from movement-caused acceleration , magnetic distortion are used to weigh the credibility of vector observation and angular rate integration. Compared with aforementioned Kalman filters and complementary filters, the fusion coefficient of our proposed algorithm is auto-tuned by the predetermined function. In addition, because the function is calculated before orientation fusion, this algorithm is computationally efficient so that it has the potential of real-time motion tracking.

## 3. Method

**Figure 1:**
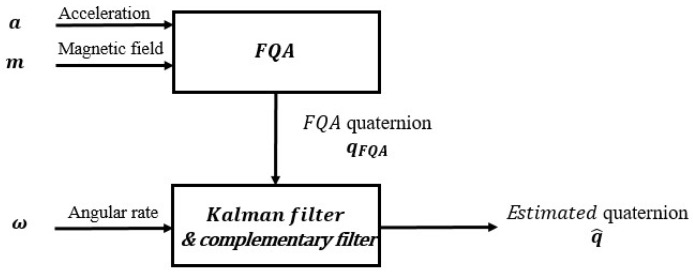
The principle block diagram of the designed filters.

As stated above, the goal of this paper is to design a computationally efficient algorithm for estimating the orientation of human-limb segments. For this goal, a dynamic estimating model that could process the data collected by IMUs should be necessarily established to represent dynamic motions of human body segments. Human musculoskeletal dynamics models which have been studied for many years are too complex to be applied in real-time motion-tracking algorithm. Hence, the challenge is to develop a simple enough model for real-time motion-tracking applications. As shown in Figure 1, measurement vectors consisting of a 3-D acceleration and a 3-D local magnetic field could be pre-processed by a geometrically intuitive motion-tracking algorithm named factored quaternion algorithm (FQA) to produce an orientation quaternion which will be called FQA quaternion. Compared with other vector observation methods and fusing local magnetic field vectors, acceleration vectors and angular rate vectors together to yield an orientation quaternion directly, using FQA to pre-processing signals significantly simplifies the filter design and obviously decreases the computational requirements [7]. In addition, the integration of quaternion derivative derived from angular rate could be fused with FQA quaternion to improve estimating accuracy.

### 3.1. Factored Quaternion Algorithm (FQA)

FQA [11] is an algorithm for estimating attitude of a single frame related to Earth-fixed frame using local magnetic field vector and acceleration vector as input. In what follows, details of orientation estimation for a single frame with arbitrary orientation will be obtained through decoupling rotation from inertial frame to sensor-fixed frame into firstly rotating about its z-axis and secondly rotating about its *y*-axis and finally about its *x*-axis.

The value of the sine and cosine of pitch angle can be expressed as:

(6) $$\sin\theta ={\bar{a}}_{x},\;\cos\theta =\sqrt{1-\sin^{2}\theta }$$

where ${\bar{a}}_{x}$ is the *x*-axis part of normalized acceleration vector and θ is the pitch angle. Then half-angle values are calculated by trigonometric half-angle formulas.

(7) $$\sin\frac{\theta }{2}=sign\left(\sin\theta \right)\sqrt{1-\cos\theta /2}$$

(8) $$\cos\frac{\theta }{2}=\sqrt{(1+\cos\theta )/2}$$

So the elevation about *y*-axis could be represented by a unit quaternion $q_{y}$.

(9) $$q_{y}=\left[\begin{array}{cccc} \cos\frac{\theta }{2} & 0 & \sin\frac{\theta }{2} & 0 \end{array}\right]$$

Similarly, the roll angle is computed as follow:

(10) $$\sin\varphi =-{\bar{a}}_{y}/\cos\theta ,\;\cos\varphi =-{\bar{a}}_{z}/\cos\theta$$

where ${\bar{a}}_{y}$ and ${\bar{a}}_{z}$ are the *y*-axis part and *z*-axis part of normalized acceleration vector, φ is the roll angle. Plugging Equation (10) into half-angle formulas, the roll quaternion is given by:

(11) $$q_{x}=\left[\begin{array}{cccc} \cos\frac{\varphi }{2} & \sin\frac{\varphi }{2} & 0 & 0 \end{array}\right]$$

The values of cosine and sine of yaw angle are determined by measured local magnetic field vector and local magnetic field reference vector, both of which are normalized in horizontal plane.

(12) $$\left[\begin{array}{c} \sin\psi \\ \cos\psi \end{array}\right]=\left[\begin{array}{cc} -m_{x} & m_{y}\\ -m_{y} & m_{x} \end{array}\right]\left[\begin{array}{c} N_{x}\\ N_{y} \end{array}\right]$$

where ${\left[m_{x},m_{y}\right]}^{\prime }$ is a unit measured local magnetic field vector projected into horizontal plane via elevation and roll quaternions. ${\left[N_{x},N_{y}\right]}^{\prime }$ is a unit local magnetic field reference vector. The azimuth quaternion is computed as follow after plugging Equation (12) into half-angle formulas.

(13) $$q_{z}=\left[\begin{array}{cccc} \cos\frac{\psi }{2} & 0 & 0 & \sin\frac{\psi }{2} \end{array}\right]$$

The orientation of rigid body is then given by:

(14) $$q=q_{z}⊗q_{y}⊗q_{x}$$

Due to deriving quaternion estimation from three Euler angles, FQA suffers from a singularity when pitch angle is ±$\frac{\pi }{2}$. In avoidance of singularity, an angle offset is adopted in the numerical implementation of FQA when cos θ < ε.

### 3.2. An Automatic Coefficient-Tuning Complementary Filter

**Figure 2:**
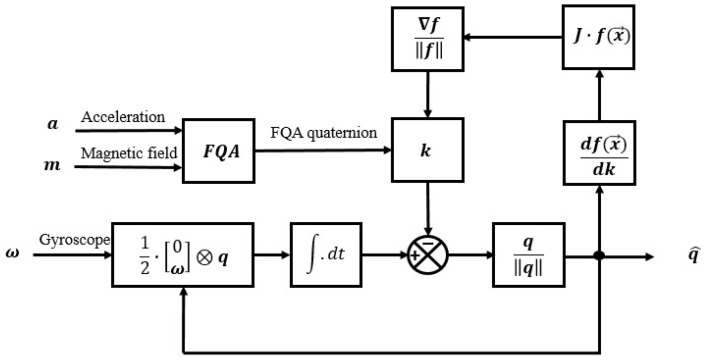
The block diagram of a complete implementation of this complementary filter.

FQA is directly used to estimate the orientation of a static or slow-moving sensor-fixed frame relative to Earth-fixed frame based on gravity vector and local magnetic field vector, which is named as the ’small movement’ assumption. However, it cannot be directly used when there is relatively large magnetic distortion and acceleration caused by dynamic movements. The accelerometer measures the sum of the gravity and the acceleration caused by movements of the attached limb. If the limb segment where an IMU is mounted is moving dynamically, the acceleration caused by movement will overwhelm the gravity. In addition, though the local magnetic field vectors are normalized in FQA, the azimuth angle it estimates still suffers from a relatively large error due to the local magnetic field distortion. This is when integration of angular rate measurements comes to help correct the orientation estimate. However, orientation estimate integrated from angular rate tends to drift over an extended period due to the integration of measurement noise. To improve the accuracy of orientation estimate, both Kalman filter and complementary filter are introduced to fuse the integrated angular rate and the FQA quaternion estimated through vector observation. Compared with Kalman filter, complementary filter is distinct by its brief equation and high computing efficiency. Complementary filter can be used to combine two estimates with different noise properties. The angular rate integration suffers from low-frequency drift while the FQA quaternion suffers from high-frequency noise brought by acceleration errors and magnetic field distortion. The equation of complementary filter is given by:

(15) $$q_{fus}=k\cdot q_{FQA}+\left(1-k\right)\cdot q_{gyro}$$

where *k* is the fusion coefficient of complementary filter. $q_{fus}$, $q_{FQA}$ and $q_{gyro}$ are fused orientation quaternion, FQA quaternion and orientation quaternion integrated from angular rate, respectively. In traditional way, fusion coefficient *k* stays consistent for all sample points after being manually tuned, which makes Kalman filter more competitive due to its capability of auto-tuning Kalman gain according to measurement and process noise variance. In this paper, fusion coefficient *k* is designed to be dynamically calculated for each sample point to weigh the credibility given to FQA quaternion and quaternion integrated from angular rate, whose principle is shown in Figure 2. Firstly, there is assumed to be a relation between fusion coefficient and the deviation of measurements and references. Tested by experiments, a linear equation for coefficient dynamic calculation is selected due to its relatively high accuracy and computing efficiency, given by:

(16) $$\begin{array}{c} k=A(\Delta_{bias}-C)+B\;A,B,C\in R\\ \Delta_{bias}=\Delta_{mag}+\alpha \Delta_{acc}\;\alpha \in \left[0,1\right] \end{array}$$

where *A* and *B* are slope and intercept of the linear equation. *C* is the threshold of computing representing the measurement of deviation extent. $\Delta_{bias}$ is the sum of the magnetic and acceleration deviation representing the errors between rotated measurement vectors and local reference vectors in the Earth-fixed frame where a factor α is used to weigh the importance of magnetic distortion and movement-caused acceleration. In addition, the $\Delta_{mag}$ and $\Delta_{acc}$ is given by:

(17) $$\Delta_{mag}=∥N-q_{FQA}⊗\left[\begin{array}{c} 0\\ m \end{array}\right]⊗q_{FQA}^{-1}∥$$

(18) $$\Delta_{acc}=∥G-q_{FQA}⊗\left[\begin{array}{c} 0\\ a \end{array}\right]⊗q_{FQA}^{-1}∥$$

where N and G are local magnetic field vector and gravitational acceleration vector in the Earth-fixed frame respectively. In addition, m and a are measured vectors of magnetic field and acceleration. To minimize $\Delta_{bias}$, an optimization method should be applied to search for optimized three factors of the linear equation. Experiments show that the iteration step of LM method is too small to find the optimized solution. In addition, Gauss-Newton algorithm suffers from large iteration amounts and low computing efficiency. This is because the best solution for minimizing $\Delta_{bias}$ should balance the influence of magnetic distortion and acceleration caused by movement. Hence, gradient descent algorithm is selected due to its simple implementation and calculation. The Equation (19) describes the iteration form for $k^{st}$ step based on an ’inertial guess’ of $x_{0}$ and a variable step size μ. The Equation (20) calculates a decent direction on the solution surface defined by the given function f(x) and its Jacobian J.

(19) $$\begin{array}{c} x_{k+1}=x_{k}+\mu_{k}\cdot \frac{\nabla f(x_{k})}{∥f(x_{k}∥)} \end{array}$$

(20) $$\begin{array}{c} \nabla f\left(x_{k}\right)=J(x_{k})f\left(x_{k}\right) \end{array}$$

(21) $$f\left(x_{k}\right)=∥N-q_{fus}⊗\left[\begin{array}{c} 0\\ m \end{array}\right]⊗q_{fus}^{-1}∥+\alpha \cdot ∥G-q_{fus}⊗\left[\begin{array}{c} 0\\ a \end{array}\right]⊗q_{fus}^{-1}∥$$

To define the Jacobian matrix,

(22) $$J(x)=\frac{df(x)}{dx}$$

The rotation represented by quaternion should be translated into rotation matrix following the formula given by:

(23) $$q⊗\left[\begin{array}{c} 0\\ j \end{array}\right]⊗q^{-1}=\left[\begin{array}{cc} 1 & 0\\ 0 & R(q) \end{array}\right]\left[\begin{array}{c} 0\\ j \end{array}\right]$$

where

(24) $$\;R\left(q\right)=\left[\begin{array}{ccc} q_{0}^{2}+q_{1}^{2}-q_{3}^{2}-q_{4}^{2} & 2q_{1}q_{2}+2q_{0}q_{3} & 2q_{1}q_{3}-2q_{0}q_{2}\\ 2q_{1}q_{2}-2q_{0}q_{3} & q_{0}^{2}-q_{1}^{2}+q_{2}^{2}-q_{3}^{2} & 2q_{2}q_{3}+2q_{0}q_{1}\\ 2q_{1}q_{3}+2q_{0}q_{2} & 2q_{2}q_{3}-2q_{0}q_{1} & q_{0}^{2}-q_{1}^{2}-q_{2}^{2}+q_{3}^{2} \end{array}\right]$$

So f(x) could be expressed as:

(25) $$f\left(x\right)=∥N-R_{\mathrm{fus}}\left(x\right)\cdot m∥+\alpha \cdot ∥G-R_{\mathrm{fus}}\left(x\right)\cdot a∥$$

The following derivative should be considered as:

(26) $$\frac{df(x)}{dk}=\frac{N-R_{\mathrm{fus}}m}{∥N-R_{\mathrm{fus}}m∥}\cdot \frac{d(N-R_{\mathrm{fus}}m)}{dR_{\mathrm{fus}}}\cdot \frac{dR_{\mathrm{fus}}}{dk}+\alpha \cdot \frac{G-R_{\mathrm{fus}}a}{∥G-R_{\mathrm{fus}}a∥}\cdot \frac{d(G-R_{\mathrm{fus}}a)}{dR_{\mathrm{fus}}}\cdot \frac{dR_{\mathrm{fus}}}{dk}$$

Thus, Jacobian matrix could be constructed as:

(27) $$J=\frac{df(x)}{dk}\cdot \frac{dk}{dx}$$

An appropriate step size $\mu_{k}$ should represent the changing rate of m and a so that an unnecessarily large step could be avoided. To do so, $\mu_{k}$ could be calculated as Equation (28) following the idea of [21] where Δt is the sample period, $\omega_{k}$ is angular rate measured by gyroscope and β is a reduction of $\mu_{k}$ to account for the noise of measured angular rate.

(28) $$\mu_{k}=\beta ∥\omega_{k}∥\Delta t$$

### 3.3. Kalman Filter Design

**Figure 3:**
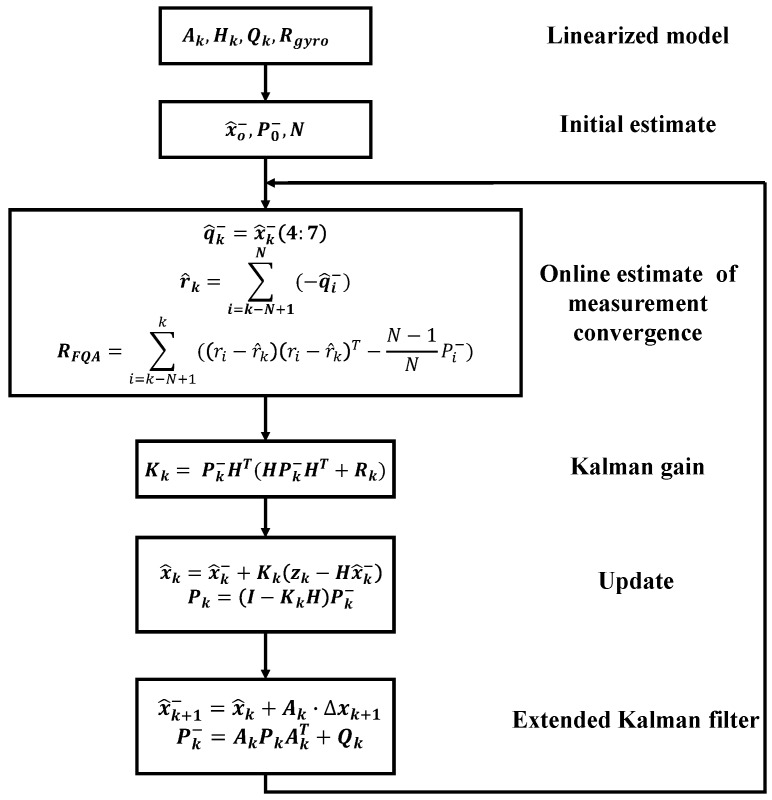
Block diagram of the adaptive Kalman filter.

To make a comparison with complementary filter designed above, an adaptive Kalman filter based on [7] is designed to fuse the integration of angular rate with FQA quaternion, the iteration progress of which is shown in Figure 3. Compared with [7] which uses QUEST to preprocess the signals from magnetometer and accelerometer, the Kalman filter we proposed introduces FQA to yield orientation estimate. In addition, the designed Kalman filter is introduced to dynamically estimate the covariance matrix of measurement.

The state vector x is a 7-D vector with the first three elements being angular rate vector and the last four elements being quaternion, which is:

(29) $$x=\left[\begin{array}{c} \omega \\ q \end{array}\right]={\left[\omega_{1},\omega_{2},\omega_{3},q_{0},q_{1},q_{2},q_{3}\right]}^{\prime }$$

The derivative of angular rate is the sum of white noise and negative angular rate divided by a time constant τ, which measures how fast a segment can move. In addition, the derivative of quaternion is derived from the multiplication of normalized quaternion $\hat{q}$ and angular rate. That is:

(30) $$\left[\begin{array}{c} {\dot{\omega }}_{1}\\ {\dot{\omega }}_{2}\\ {\dot{\omega }}_{3} \end{array}\right]=\frac{1}{\tau }\cdot \left(-\left[\begin{array}{c} \omega_{1}\\ \omega_{2}\\ \omega_{3} \end{array}\right]+\left[\begin{array}{c} w_{1}\\ w_{2}\\ w_{3} \end{array}\right]\right)$$

(31) $$\left[\begin{array}{c} {\dot{q}}_{0}\\ {\dot{q}}_{1}\\ {\dot{q}}_{2}\\ {\dot{q}}_{3} \end{array}\right]=\frac{1}{2}\left[\begin{array}{c} {\hat{q}}_{0}\\ {\hat{q}}_{1}\\ {\hat{q}}_{2}\\ {\hat{q}}_{3} \end{array}\right]\cdot \left[\begin{array}{c} 0\\ \omega_{1}\\ \omega_{2}\\ \omega_{3} \end{array}\right]$$

The state equation could be derived by linearizing Equation (32) using its first-order Taylor expansion and integrate the small increment by sample period Δt into actual state vector, given by:

(32) $$\begin{array}{c} \Delta x^{k+1}=A_{k}\Delta x^{k}+w^{k}\\ A_{k}=\left[\begin{array}{cc} e^{\frac{-\Delta t}{\tau }}\cdot I_{3} & 0_{3\times 4}\\ D_{k} & I_{4} \end{array}\right]\\ D_{k}=\left[\begin{array}{ccc} -x_{5}^{k}\cdot \Delta t & -x_{6}^{k}\cdot \Delta t & -x_{7}^{k}\cdot \Delta t\\ x_{4}^{k}\cdot \Delta t & x_{7}^{k}\cdot \Delta t & -x_{6}^{k}\cdot \Delta t\\ -x_{7}^{k}\cdot \Delta t & x_{4}^{k}\cdot \Delta t & x_{5}^{k}\cdot \Delta t\\ x_{6}^{k}\cdot \Delta t & -x_{5}^{k}\cdot \Delta t & x_{4}^{k}\cdot \Delta t \end{array}\right] \end{array}$$

where $w^{k}$ is the integration of noises in angular rate. Since angular rate and FQA quaternion are parts of the state vector, the measurement vector of Kalman filter could be simply given as the following:

(33) $$z^{k}=H_{k}x^{k}+v^{k}$$

where $H_{k}$ is a 7×7 identity matrix. As what we designed, $D_{k}$, $H_{k}$, covariance matrix of process and measurement noises $Q_{k}$ and $R_{k}$ should be provided to start the designed Kalman filter.

According to the definition of covariance matrix of process noise,

(34) $$Q_{k}=E\left({w^{k}}^{\prime }w^{k}\right)$$

here *E* is the expectation operator, the process noise covariance matrix $Q_{k}$ is written as such:

(35) $$Q_{k}=\left[\begin{array}{cc} \frac{D}{2\tau }\left(1-e^{\frac{\Delta t}{\tau }}\right)\cdot I_{3} & 0_{3\times 4}\\ 0_{4\times 3} & 0_{3\times 3} \end{array}\right]$$

where D is the variance of angular rate noise ${\left[w_{1},w_{2},w_{3}\right]}^{\prime }$. By matching the simulated angular rates with measured ones, D and τ could be experimentally determined.

(36) $$R_{k}=\left[\begin{array}{c} R_{gyro}^{k}\\ R_{FQA}^{k} \end{array}\right]$$

where $R_{gyro}^{k}$ is the covariance matrix of measured angular rate and $R_{FQA}^{k}$ is the covariance matrix of FQA quaternion. $R_{gyr}^{k}$ relates to the characteristics of gyroscope. Assuming the noise of gyroscope measurement is independent white noise, its variance could be experimentally determined through method stated above. Due to the unknown magnetic distortion and movement-caused acceleration, $R_{FQA}^{k}$ could be estimated online by the residual in quaternion measurement update $r_{k}=-{\hat{q}}_{k}$ where ${\hat{q}}_{k}$ is the normalized quaternion updated by state equation. Given *N* consecutive observations from i=k−N+1 to i=k, an unbiased estimate of the mean is given by:

(37) $${\hat{r}}_{k}=\frac{1}{N}\sum_{i=N-k+1}^{k}r_{i}$$

The unbiased estimation of $R_{FQA}^{k}$ can be obtained as Equation (38), following the method of [25].

(38) $$R_{FQA}^{k}=\sum_{i=k-N+1}^{k}\left(\left(r_{i}-{\hat{r}}_{k}\right){\left(r_{i}-{\hat{r}}_{k}\right)}^{\prime }-\frac{N-1}{N}P_{i}^{-}\right)$$

## 4. Experiment

### 4.1. Data Acquisition

While FQA works well for slow movements and nonferrous conditions, the objective of these two filters we designed is to blend gyroscope measurements and the estimates produced by accelerometer and magnetometer data when the sensor module is subject to a large linear acceleration and magnetic distortion. To this end, a 4-DOF test machine is designed to provide true data for validation. This 4-DOF machine embeds four high precision Hall sensors ($0.00024^{\circ }$ resolution, Millay Technology (https://shop108505223.taobao.com/?spm=a1z10.1-c.0.0.5f972d91mjPCyv) GT-B) that measures rotations on each axis representing three Euler angles, which are connected by four couplings (Suwei Machinery (https://suweiptc.taobao.com/?spm=2013.1.1000126.4.324e50284gMl7V) SPC-25-20-10). The measured Euler angles will be translated into quaternions to avoid the singularity of Euler angles. To make an accurate reference, these Hall sensors are immune to magnetic distortion and large acceleration. One IMU (Delsys Trigno IM, sampling time 0.0135 s, sensor characteristics available at https://www.delsys.com/support/trigno-im/) is rigidly mounted on the second DOF to avoid the errors caused by axes’ cross-coupling. In addition, the sensor was initially placed with its axis aligned with north-east-down directions. The resulting apparatus enables the direct comparison of IMU-estimated orientation to values measured by Hall sensors over a wide range of 3-D movement.

**Figure 4:**
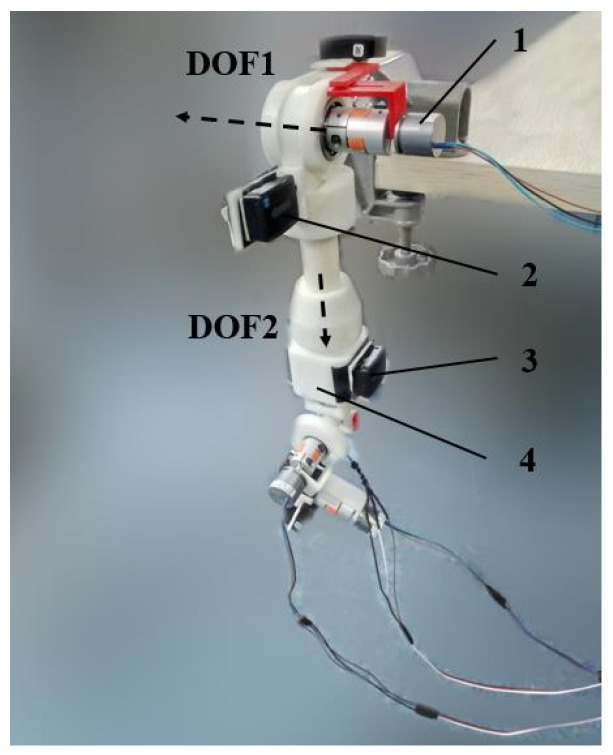
Experimental setup using a 4 DOF test machine. 1—Hall sensor that measures the rotation angle of DOF1, 2—IMU1 beside DOF1, 3—IMU1 beside DOF2, 4—Hall sensor embedded in the test machine that measures the rotation angle of DOF2. Dashed lines represent the axes of DOF1 and DOF2 respectively.

Data from the IMU are synchronized with the Hall sensor data by rotating the first two axes of the machine (shown in Figure 4) with other axes locked. In addition, all the data after rotating about the first axis is synchronized by time labels.

**Figure 5:**
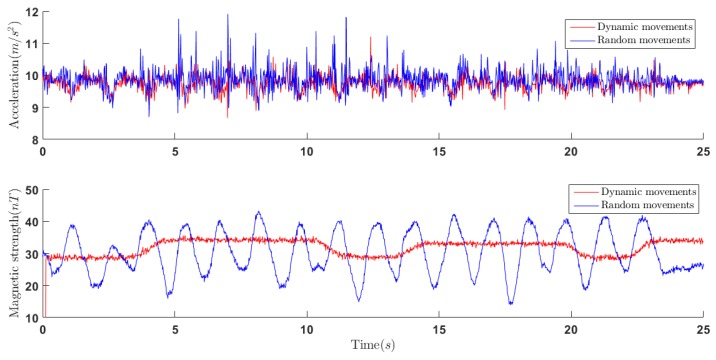
Norms of acceleration and magnetic field vectors.

To test the performance of designed algorithm while distorted local magnetic field and dynamic movement, the experiment was implemented with relatively large linear acceleration and a moving ferrous object near the sensor. It is shown in Figure 5 that the local magnetic distortion caused by moving ferrous object and the large acceleration caused by dynamic movement are presented in the readings of magnetometer and accelerometer. As shown in Figure 5, the maximum of the norm of acceleration vector is 12 m/s${}^{2}$ approximately that is more than that of local gravity vector. In addition, the magnetic strength is obviously beyond normal strength of local magnetic field, which represents local magnetic field is influenced by moving ferrous object.

### 4.2. Results and Discussion

After initializing the parameters of designed filters, both complementary filter and Kalman filter were implemented through MATLAB to test the performance and accuracy of the quaternion-based orientation estimates. In addition, for the purpose of comparison, four complementary filter-based algorithms, including an algorithm which uses gradient descent algorithm (GDA) to compute the direction of gyroscope measurement error [21], a fast complementary filter whose fusion coefficients should be pre-tuned manually (FCF) [20] and an improved algorithm using Gauss-Newton method (GN) to optimize the increment of quaternion-based orientation at each sample point [18], are also introduced to estimate the orientation of sensor-fixed frame. In addition, the Kalman filter-based algorithm (DEL) incorporated in Delsys data processing software, EMG works, is used as a reference for comparing our algorithm with commercially used algorithm. Elements of the function of fusion coefficient *k* are determined by the gradient descent algorithm as stated in Section 3. In addition, the parameters of Kalman filter are determined by minimizing the error convergence $P_{k}$ [7].

#### 4.2.1. Accuracy Analysis

**Figure 6:**
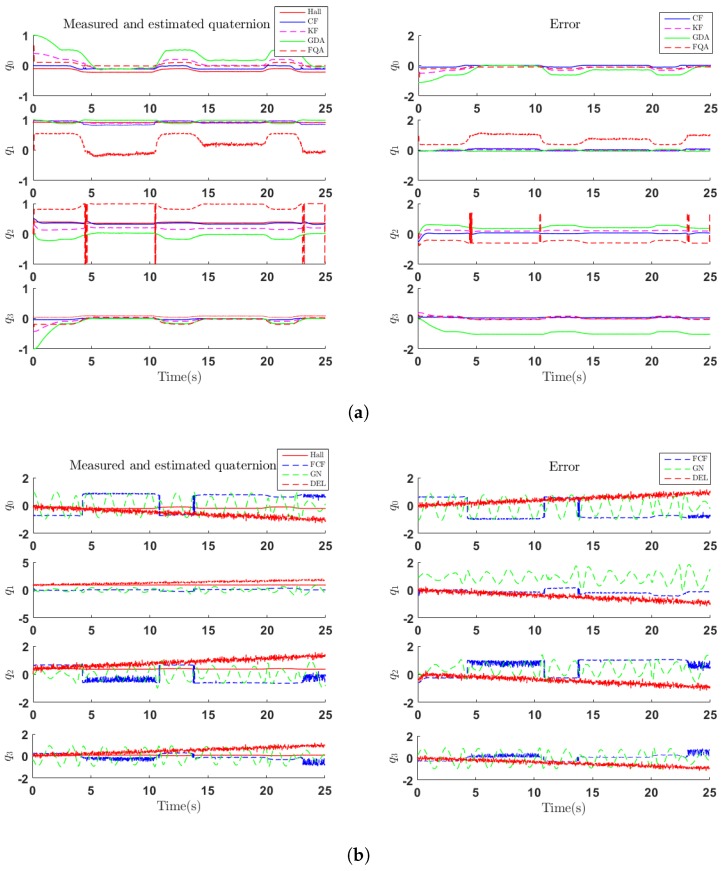
Measured and estimated quaternion (left) and error (right) of dynamic movement. (**a**) Comparison among quaternions estimated by CF, KF, GDA, FQA algorithms and reference quaternions obtained from Hall sensor. (**b**) Comparison among quaternions estimated by FCF, GN, DEL algorithms and reference quaternions obtained from Hall sensor.

In the first set of experiments, the first DOF of test machine was rotated about its axis for three times to validate the dynamic response of designed filters. The performance is evaluated by quaternions which include $q_{0}$, $q_{1}$, $q_{2}$, $q_{3}$ elements of the orientation. In Figure 6, the graph to the left shows the comparison of measured quaternion of first DOF (Hall) and quaternion estimated by the complementary filter designed in this paper (CF), GDA algorithm (GDA), Kalman filter (KF), FQA algorithm (FQA), FCF algorithm (FCF), GN algorithm (GN) and the algorithm embedded in Delsys (DEL). In addition, the graphs to the right show the error of aforementioned algorithms. It can be seen that GDA algorithm suffers from a slow convergence procedure which will contribute to a large error in the start of the algorithm. Compared with GDA and CF algorithms, GN and FCF, which are also complementary filters, suffer from much more errors and oscillations, which refers to the dramatic oscillations of acceleration and magnetic strength. This reflects the importance of dynamically weighing the credibility of each signal through dynamically tuning the fusion coefficient *k*. To evaluate the superiority of the designed filter, we discarded the starting 2.5 s of our data, which is related to the convergence process of GDA algorithm and Kalman filter, to evaluate errors of each algorithm.

**Figure 7:**
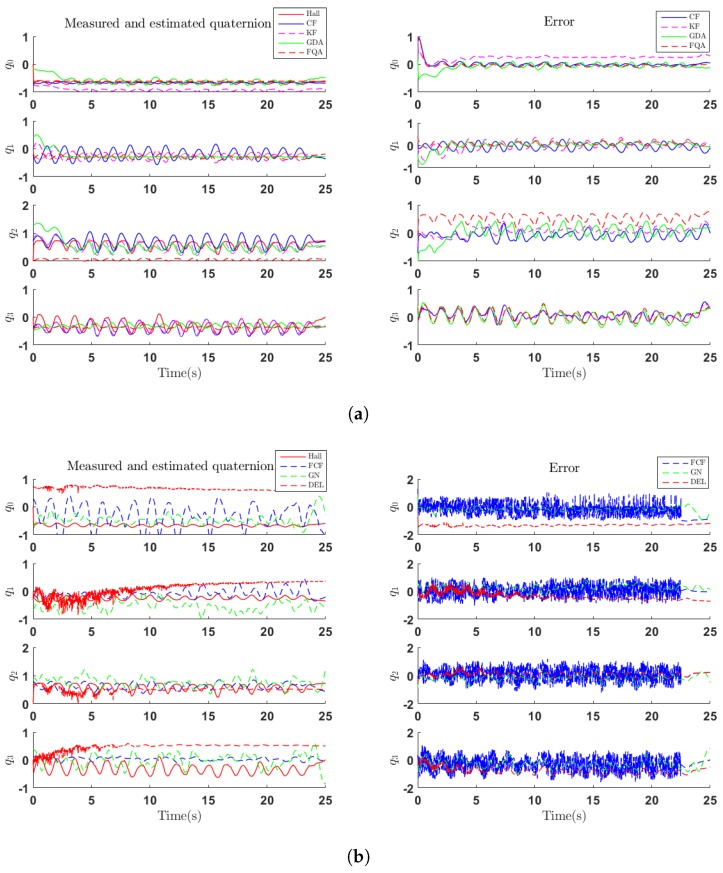
Measured and estimated quaternion (left) and error (right) of random movement. (**a**) Comparison among quaternions estimated by CF, KF, GDA, FQA algorithms and reference quaternions obtained from Hall sensor. (**b**) Comparison among quaternions estimated by FCF, GN, DEL algorithms and reference quaternions obtained from Hall sensor.

Although dynamic movements are performed for validation, the other set of experiments should be conducted to validate the performance under "free movement" conditions. In the second set of experiments, random movements are performed in which sensors are mounted on the second DOF of test machine. Figure 7 shows the performance of aforementioned algorithms in estimating the orientation of sensor-fixed frame, respectively. The performance of each algorithm is represented by quaternions in each time sample where $q_{0}$, $q_{1}$, $q_{2}$, $q_{3}$ represent elements of a quaternion at each time sample. The red lines in each graph represent the quaternions measured by Hall sensors (Hall). In Figure 7a, the graphs to the left show the orientation estimated by CF, KF, GDA and FQA algorithms, and the graphs to the right show the errors of these algorithms. It can be seen that FQA brings a lot of high-frequency noise into orientation estimate. As shown in Figure 7a, both KF and GDA algorithm present a descent progress which refers to their convergence procedure. Figure 7b shows that GN and FCF algorithm still suffer from a relatively large oscillation in random movements due to the corrupted acceleration and magnetic field. In addition, DEL algorithm, although with little oscillation, suffers from a relatively large offset.

RMS (Root-Mean-Square) error is used to measure the deviation of every algorithm with respect to the measurement of Hall sensors. The RMS errors of aforementioned algorithms are shown in Table 1 and Table 2 where the adjustable factors of CF and GDA algorithm are 1.5 and 0.2, respectively. It could be seen that the performance of CF algorithm and KF algorithm is both better than that of GDA algorithm. Compared with RMS errors of CF algorithm, KF algorithm suffers from higher RMS errors in each quaternion element due to its linearized process model and its adaptive estimating of $R_{FQA}$. The RMS errors of FQA algorithm are obviously higher than RMS errors of CF and KF algorithms, which demonstrates the effectiveness of introducing integrated angular rate into orientation estimate. The RMS errors of GN and FCF algorithms are much higher than other algorithms, which reflects the effectiveness of introducing vector observation method. To test the superiority of the algorithms we proposed against commercially available algorithms, the RMS errors of DEL shown in Table 1 demonstrates the better accuracy of CF and KF algorithms. And it is demonstrated in [21] that GDA algorithm has a better performance compared with the Kalman filter-based orientation estimating algorithm incorporated in the accompanying software of Xsens MTx orientation sensor, which provides a side proof of the superiority of CF algorithm.

#### 4.2.2. Computational Efficiency and Stability Analysis

To make a comparison of computational efficiency, the computational time of executing each algorithm is calculated by clock function of MATLAB when these algorithms are used to process data of 1850 sample points lasting 25 s . As shown in Table 3, computational time required to execute the CF algorithm is 0.57995 s, which is the least among the executing time of all aforementioned data-fusing algorithms. FQA algorithm is excluded because it is embedded into KF and CF algorithms. Due to the execution of Gauss-Newton method at each sample point, GN algorithm requires the most computational time. KF algorithm also suffers from a relatively low computational efficiency which demonstrates the complicated iteration progress of Kalman filter as shown in Figure 3. Although the fusion coefficient of FCF algorithm is tuned manually, which reduces its complexity, its relatively large amount of matrix calculations still contributes to an increasing computational efficiency. Compared with other algorithms, it can be seen that CF algorithm demonstrates the largest potential of real-time motion tracking.

**Figure 8:**
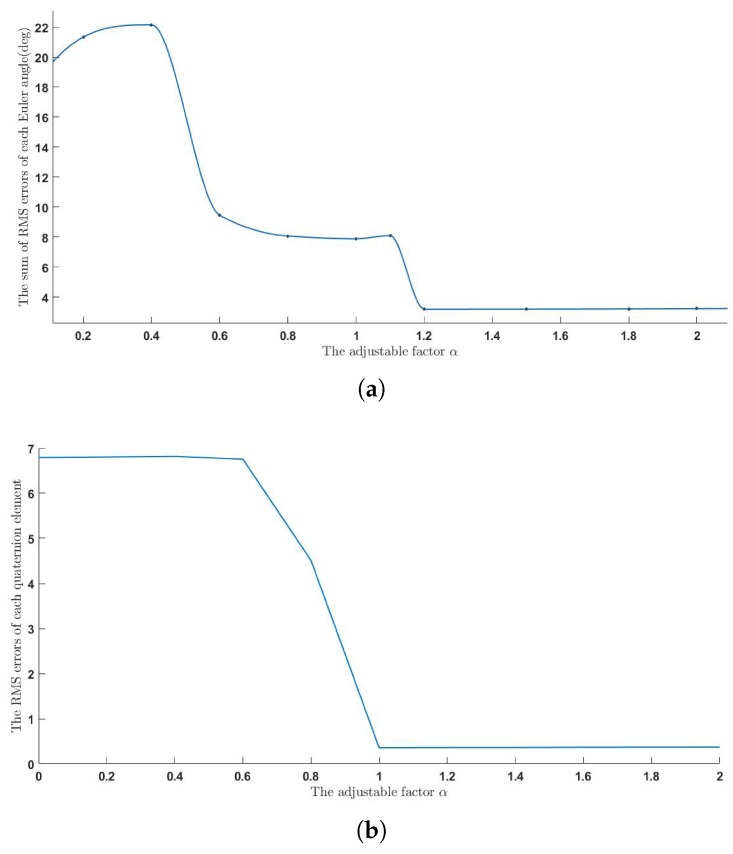
The effect of adjustable factor α. (**a**) The effect of adjustable factor α in dynamic movements. (**b**) The effect of adjustable factor α in random movements.

To measure the parameter sensitivity of the complementary filer we designed, the relationship between the factor α, which weighs the importance of magnetic distortion and movement-caused acceleration in complementary filter, and the sum of RMS errors is shown in Figure 8. It is shown that the importance of movement-caused acceleration weighs more than the importance of magnetic distortion. In addition, when α is more than 1.2, the performance of our algorithm is not sensitive to this parameter.

The stability issue of CF algorithm includes the convergence of the fusion coefficient function *k* and the avoidance of introducing divergence caused by angular rate integration. The convergence of *k* depends on the number of sample points used to train this function. Experiments show that the convergence of *k* can still be guaranteed when the number of sample points was reduced to 20. After obtaining the fusion coefficient function, the stability of orientation estimating progress only refers to the divergence caused by angular rate integration. If the fusion quaternion $q_{fus}$ is used as the quaternion of last moment $q_{t-1}$ in Equation (1), the noise of gyroscope readings will be accumulated so that the orientation estimate will be divergent. To solve this problem, FQA quaternion $q_{FQA}$ of last moment is used in Equation (1) to predict the quaternion $q_{t}$ which is then fuse with FQA quaternion of this moment.

## 5. Conclusions

We have considered the standard sensor fusion of orientation estimate. In addition, a coefficient auto-tuning complementary filter has been designed by highlighting the malicious effects of inhomogeneous magnetic field and movement-caused acceleration. Its coefficient is auto-tuned by a predetermined univariate function. In this function, the independent variable is the bias between magnetometer and accelerometer measurements rotated by FQA quaternion and their reference vectors. In addition, the factors of this function are predetermined by a gradient descent method which could be iterated before performing orientation estimating algorithm. After determining the coefficient tuning function, the Euler angle-based orientation estimated by FQA could be dynamically fused with the quaternion-based orientation integrated from angular rate. To validate the performance of the algorithm, experimental results tested by a 4-DOF testing machine is presented to make a comparision with an adaptive extended Kalman filter and other four complementary filter-based algorithms. To make a comparison with commercially available algorithms, we test the performance of the algorithm embedded in Delsys data processing software, while an indirect comparison with Xsens is also included. As shown in Section 4, the accuracy and computational time period of the algorithm we designed are the best, which demonstrates its suitability for many applications of inertial sensors including human motion analysis.

In this paper, the magnetic distortion we introduced in our experiments is caused by some ferromagnetic objects which moved in a relatively regular way. Under this condition, the algorithm we proposed could compensate the malicious effects of inhomogeneous magnetic field. The performance of our algorithm might decrease when more ferromagnetic objects are placed around. In addition, some sample points are needed to train a fusion coefficient function, which means orientations at these sample points cannot be estimated in real time. To solve the drawback of our algorithm stated above, a more stable and computationally efficient algorithm is required to train the function so that a fast iteration of this function could be achieved using fewer sample points when magnetic distortion varies rapidly.

Our future work is concerned with combining this work with sensor-to-body alignment method, such as [26,27], to calculate kinematics of human lower-limb joints, estimate the trajectory of human segments and make a validation against optical motion capture system.

## Figures and Tables

**Table 1:** RMS errors of dynamic movement.

Algorithm	CF	KF	GDA	FQA	FCF	GN	DEL
RMS error $q_{0}$	0.0312	0.0465	0.2208	0.0622	0.3762	0.3653	0.2958
RMS error $q_{1}$	0.0333	0.0558	0.0351	0.5414	0.2673	0.9790	0.3093
RMS error $q_{2}$	0.0534	0.0458	0.2124	0.3440	0.3805	0.3348	0.2709
RMS error $q_{3}$	0.0371	0.0678	0.9070	0.0911	0.2369	0.3329	0.3217

**Table 2:** RMS errors of random movement.

Algorithm	CF	KF	GDA	FQA	FCF	GN	DEL
RMS error $q_{0}$	0.0372	0.0880	0.0561	0.1511	0.2088	0.1551	1.3173
RMS error $q_{1}$	0.0852	0.1034	0.1631	0.1333	0.7500	0.9869	0.4463
RMS error $q_{2}$	0.0659	0.0975	0.0596	0.1361	0.7846	1.0760	0.1675
RMS error $q_{3}$	0.1774	0.3525	0.2866	0.4157	0.1127	0.6512	0.8094

**Table 3:** Computational time of executing each algorithm.

Algorithm	CF	KF	GDA	FCF	GN
Computational time (s)	0.57995	0.91342	0.68451	0.6344	1.1746

## Abbreviations

The following abbreviations are used in this manuscript:

IMU	Inertial Measurement Unit
DOF	Degree of freedom
FQA	Factored quaternion algorithm
GDA	Orientation estimating algorithm using a gradient descent algorithm
CF	Complementary filter proposed by this paper
KF	Kalman filter improved by this paper
FCF	A fast complementary filter whose fusion coefficients should be pre-tuned manually
GN	An improved algorithm using Gauss-Newton method

## References

[1] Zhou H., Hu H. (2008). Human motion tracking for rehabilitation—A survey. Biomed. Signal Process. Control.

[2] Nair L.H. AHRS based body orientation estimation for real time fall detection. Proceedings of the International Conference on Innovations in Information, Embedded and Communication Systems.

[3] Yang U.J., Kim J.Y. (2015). Mechanical design of powered prosthetic leg and walking pattern generation based on motion capture data. Adv. Robot..

[4] Gu X., Zhang Y., Sun W., Bian Y., Zhou D., Kristensson P.O. Dexmo: An Inexpensive and Lightweight Mechanical Exoskeleton for Motion Capture and Force Feedback in VR. Proceedings of the 2016 CHI Conference on Human Factors in Computing Systems.

[5] Shimizu M., Koide K., Ardiyanto I., Miura J., Oishi S. LIDAR-based body orientation estimation by integrating shape and motion information. Proceedings of the 2016 IEEE International Conference on Robotics and Biomimetics (ROBIO).

[6] Lamine H., Bennour S., Laribi M.A., Romdhane L., Zaghloul S. (2017). Evaluation of Calibrated Kinect Gait Kinematics Using a Vicon Motion Capture System. Comput. Methods Biomech. Biomed. Eng..

[7] Yun X., Bachmann E.R. (2006). Design, Implementation, and Experimental Results of a Quaternion-Based Kalman Filter for Human Body Motion Tracking. IEEE Trans. Robot..

[8] Luinge H.J., Veltink P.H. (2004). Inclination measurement of human movement using a 3-D accelerometer with autocalibration. IEEE Trans. Neural Syst. Rehabil. Eng..

[9] Ligorio G., Sabatini A.M. (2015). A Novel Kalman Filter for Human Motion Tracking with an Inertial-Based Dynamic Inclinometer. IEEE Trans. Biomed. Eng..

[10] Shuster M.D., Oh S.D. (1981). Three-axis attitude determination from vector observations. J. Guid. Control Dyn..

[11] Yun X., Bachmann E.R., McGhee R.B. (2008). A Simplified Quaternion-Based Algorithm for Orientation Estimation From Earth Gravity and Magnetic Field Measurements. IEEE Trans. Instrum. Meas..

[12] Phan D., Kashyap B., Pathirana P.N., Seneviratne A. A constrained nonlinear optimization solution for 3D orientation estimation of the human limb. Proceedings of the 2017 10th Biomedical Engineering International Conference (BMEiCON).

[13] Makni A., Fourati H., Kibangou A.Y. Adaptive Kalman filter for MEMS-IMU based attitude estimation under external acceleration and parsimonious use of gyroscopes. Proceedings of the 2014 European Control Conference (ECC).

[14] Sabatini A.M. (2011). Estimating Three-Dimensional Orientation of Human Body Parts by Inertial/Magnetic Sensing. Sensors.

[15] Roetenberg D., Luinge H.J., Baten C.T.M., Veltink P.H. (2005). Compensation of magnetic disturbances improves inertial and magnetic sensing of human body segment orientation. IEEE Trans. Neural Syst. Rehabil. Eng..

[16] Roetenberg D., Baten C.T.M., Veltink P.H. (2007). Estimating Body Segment Orientation by Applying Inertial and Magnetic Sensing Near Ferromagnetic Materials. IEEE Trans. Neural Syst. Rehabil. Eng..

[17] Roetenberg D., Luinge H., Slycke P. Xsens MVN: Full 6DOF Human Motion Tracking Using Miniature Inertial Sensors. http://www.xsens.com/images/stories/PDF/MVNwhitepaper.pdf.

[18] Bachmann E.R., McGhee R.B., Yun X., Zyda M.J. (2001). Inertial and Magnetic Posture Tracking for Inserting Humans into Networked Virtual Environments. Proceedings of the ACM Symposium on Virtual Reality Software and Technology.

[19] Gallagher A., Matsuoka Y., Ang W.T. An efficient real-time human posture tracking algorithm using low-cost inertial and magnetic sensors. Proceedings of the 2004 IEEE/RSJ International Conference on Intelligent Robots and Systems (IROS) (IEEE Cat. No.04CH37566).

[20] Wu J., Zhou Z., Chen J., Fourati H., Li R. (2016). Fast Complementary Filter for Attitude Estimation Using Low-Cost MARG Sensors. IEEE Sens. J..

[21] Madgwick S.O.H., Harrison A.J.L., Vaidyanathan R. Estimation of IMU and MARG orientation using a gradient descent algorithm. Proceedings of the 2011 IEEE International Conference on Rehabilitation Robotics.

[22] Seel T., Ruppin S. (2017). Eliminating the Effect of Magnetic Disturbances on the Inclination Estimates of Inertial Sensors. IFAC-PapersOnLine.

[23] Zhang S., Jin W., Zhang Y. Implementation and complexity analysis of orientation estimation algorithms for human body motion tracking using low-cost sensors. Proceedings of the 2017 2nd International Conference on Frontiers of Sensors Technologies (ICFST).

[24] Yadav N., Bleakley C. (2014). Accurate Orientation Estimation Using AHRS under Conditions of Magnetic Distortion. Sensors.

[25] Myers K., Tapley B. (1976). Adaptive sequential estimation with unknown noise statistics. IEEE Trans. Autom. Control.

[26] Palermo E., Rossi S., Marini F., Patanè F., Cappa P. (2014). Experimental evaluation of accuracy and repeatability of a novel body-to-sensor calibration procedure for inertial sensor-based gait analysis. Measurement.

[27] Vargas-Valencia L.S., Elias A., Rocon E., Bastos-Filho T., Frizera A. (2016). An IMU-to-Body Alignment Method Applied to Human Gait Analysis. Sensors.

